# Regeneration in planarians modifies behavioral switching

**DOI:** 10.1016/j.isci.2025.111947

**Published:** 2025-02-03

**Authors:** Nayana S. Tellakula, Eva-Maria S. Collins, William B. Kristan

**Affiliations:** 1 Department of Neurobiology, School of Biological Sciences, University of California, San Diego, La Jolla, CA, USA; 2 Department of Biology, Swarthmore College, Swarthmore, PA, USA; 3 Department of Neuroscience, Perelman School of Medicine, University of Pennsylvania, Philadelphia, PA, USA; 4 Department of Physics and Astronomy, Swarthmore College, Swarthmore, PA, USA

**Keywords:** Biological sciences, Developmental anatomy, Histology

## Abstract

The planarian *Dugesia japonica* responds differently to localized stimuli: anterior regions turn, middle regions elongate, and posterior regions contract. If cut into several pieces, each piece immediately produces the same three responses. Over several days, each piece regenerates all transected body parts. This study tested how the pieces coordinate behavioral responses during regeneration. We first determined the locations of the turning/elongation and elongation/contraction behavioral switches. Immediately, all transections moved both switching sites away from the cut sites so that the worm pieces produced the same three responses as intact worms. During regeneration, the sites of behavioral switching moved progressively closer to the transection (now regeneration) sites. These results show that the immediate effects of transection (likely physiological) are coordinated with the addition of regenerating tissue (anatomical) to maintain as normal an animal as possible. Other animals that regenerate body parts, such as amphibians and reptiles, may use similar coordination mechanisms.

## Highlights

• Planarians turn (front), elongate (middle), or contract (back) to local stimuli
• Switching between turn-elongate and elongate-contract occurs at specific regions
• Switch locations move away from the cut sites in bisected worms to restore the behaviors
• Because switch locations change dynamically, regenerates behave like intact worms

## Graphical abstract

**Figure:**
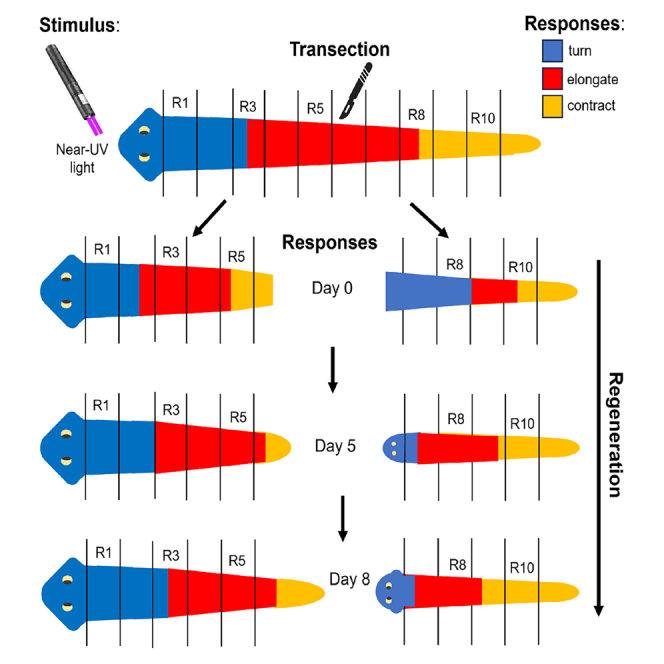

## Introduction

Planarians can regenerate any portion of their body that is removed by their own behavior, by injury, or by surgical transection.^1^^,^^2^ Intact *Dugesia japonica* planarians exhibit three distinct behaviors in response to a mild mechanical or near-UV light stimulus at different body regions: turning (head), elongation (middle), and contraction (tail).^3^ Transecting *D. japonica* at different locations showed that pieces of a planarian behaved like intact worms.^3^ If both the anterior and posterior portions of a planarian are removed, for instance, the remaining middle portion—which before the transections would produce only elongation—immediately produces all three behaviors in the normal order: turning to anterior stimuli, elongation to midbody stimuli, and contraction to posterior stimuli, even in a piece of a worm that in the intact worm would produce only one of the behaviors. We wanted to determine how regeneration would affect the interactions among these behavioral responses. To do so, we first studied how regeneration affects the individual responses and then determined which specific body region was the site of switching from one behavior to another. Finally, we transected worms at different locations and followed changes in this site during regeneration. We found that the switching site did change over the course of 8 days in a way that preserved many of the features seen in intact worms.

## Results

### Locations of the regions of behavioral switching, defining 12 body regions

**Figure 1:**
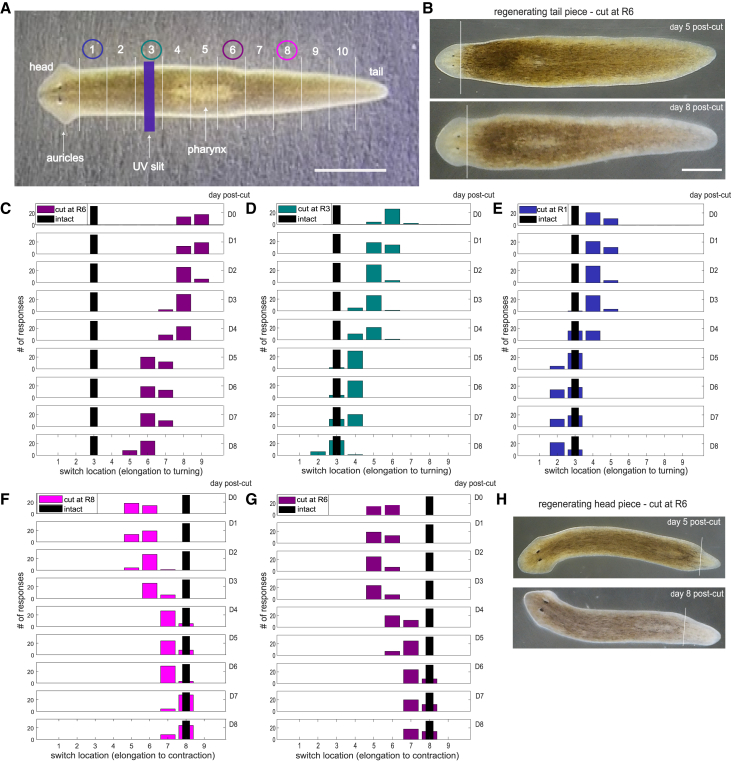
The locations of behavioral switching change during regeneration (A) Locations of the regions of behavioral switching, defining 12 body regions. Picture of an intact planarian with 12 regions indicated: head plus 11 body regions of equal length, one of which is the tail. The purple bar represents one location of a near-UV slit stimulus. Colored circles show the transection sites for the experiments corresponding to (C–G). Scale bar, 2 mm. (B) Pictures of a planarian tail piece resulting from transection at region 6, on day 5 (upper) and day 8 (lower) after transection. The tissue to the left of the white lines was regenerating tissue, indicated by the absence of dark pigmentation. The regions in the transected planarians retained the designations assigned to them in intact worms. For instance, the posterior end of worms transected at region 6 consisted of regions 6–10 and a tail. The regenerative tissue forming anterior to the cut site was designated region 5. Scale bar, 0.5 mm for both images. (C–E) Locating regions of switching in regenerating posterior pieces. Graphs show the region of behavioral switching from elongation to turning in the posterior pieces of planarians for transections at regions 6 (C), 3 (D), and 1 (E). Note that for a few cases on day 8 in the data of (D) and (E), the site of switching was in the regenerating tissue; accordingly, these were assigned to region 5 (D) and region 2 (E). (F and G) Locating regions of switching in regenerating anterior pieces. Graphs show the region of behavioral switching from elongation to contraction in the posterior pieces for transections at regions 8 (F) and 6 (G). In (F), many of the behavioral switches were observed in the regenerating tissue and therefore were assigned to regions 7 or 8, depending on how far it was away from the original cut site. Every graph used a different set of 16 planarians, each of which was stimulated twice on each of the 9 days for a total of 32 responses. Because our data were not normally distributed, we used mixed-effects ordinal logistic regression to account for the ordered values of the locations that behavioral switches occurred along the planarian, while accounting for random effects of individual planarian variability. For these measures, we used R version 4.4.2 with the “clmm” function from the “ordinal” package to model the ordered nature of the behavior response. We then used post hoc Tukey tests to compare differences between days within each cut location condition, using the “emmeans” package. We used location along the planarian as the ordinal response variable and the day of regeneration as a numeric predictor. Cut location was a categorical predictor. The method looked at the interaction between day and cut location to evaluate whether the effect of day post amputation varied across cut location conditions. There is a significant interaction between day number and cut location, so we did post hoc comparisons between days within each cut location condition. Since day was used as a numeric variable, we specified the numeric values at which the post hoc comparisons should be made, using day set to a value of 1, 2, 3, 4, 5, 6, 7, 8, and 9. Tukey comparisons of all different days against each other for each cut location condition were all significant with values of *p* < 0.0001. Hence, the switch location measured for each day after a given transection was strongly significantly different from the values for all other days. The black bars in each graph indicate the region of switching in intact planarians. (H) Pictures of a planarian head piece resulting from transection at region 6 on day 5 (upper) and day 8 (lower) after transection. The tissue to the right of the white lines was regenerating tissue, indicated by the absence of dark pigmentation. Scale bar shown in (B) (0.5 mm) applies to both of these images.

To study how regeneration affects the three behavioral responses, we first determined which specific body regions were the sites of switching from one behavior to another. To localize the regions with different behavioral responses, we devised a metric that allowed us to compare worms of different lengths. We divided the length of each planarian into a head and 11 regions of equal length, consisting of 10 body regions and a tail (Figure 1A). The head region is distinct with eyes and auricles, and the middle region is marked by an eversible feeding tube (pharynx), spanning regions 4–6. To locate the sites of behavioral switching in intact worms, we shone a near-UV slit^3^ on a middle region that produced elongation. We then moved the slit anteriorly to determine the site at which the behavior switched to turning and posteriorly to find where the response switched to contraction. Elongation reliably switched to turning in the pre-pharyngeal region 3 (black bars in Figures 1C–1E), and elongation switched to contraction in the post-pharyngeal region 8 (black bars in Figures 1F and 1G).

### Locating regions of switching in regenerating posterior pieces

We tested posterior pieces that had been surgically transected at one of three different locations: regions 6, 3, and 1 (circled numbers in Figure 1A). On the day of transection (day 0) at region 6, the back ends switched from elongation to turning in regions 8 and 9 (Figure 1C). The distance from the cut end to the switch site was 2–3 regions, comparable to the distance from the head to the turn-inducing region in intact planarians. The elongation/turning switch site moved anteriorly over the next week as the worm regenerated its head (Figure 1B). By day 8, this switch occurred at region 6, the transection site, which is different from the switching site (region 3) in an intact worm. Hence, by day 8, the transection site was still influencing switching as though it were a head.

In planarians transected at region 3, the switch occurred at region 6 on day 0 (Figure 1D). Once again, for 8 days, the switch site moved anteriorly along the worm as it regenerated its head. In this case, the switch site returned to the original region 3 by day 8, implying that the regenerating head had gained the function of a normal head in these worms, maintaining a 3-region separation between it and the site of behavioral switching. Transecting worms at region 3 (Figure 1D) produced comparable results, except that the switching site started two regions closer to the transection site and reached the region 3, the site of switching in the intact worm, by day 8.

In planarians transected at region 1, just posterior to the auricles, the behavioral switch moved just one region posteriorly to region 4 on day 0, and by day 8, it had returned to region 2/3 (Figure 1E). The results on all three types of posterior pieces show a similar rate of behavioral recovery during regeneration: the switch site moves 2–3 regions anteriorly over the course of 8 days of regeneration.

### Locating regions of switching in regenerating anterior pieces

We then asked whether the anterior portion of transected planarians (the “head end”) showed similar changes in the site of switching as the tail end regenerated. To address this question, we transected planarians at two different locations (post-pharyngeal regions 8 and 6) and determined the site of the elongation/contraction switch as the posterior end regenerated (Figure 1H). Intact *D. japonica* showed this behavioral switch at region 8 (Figures 1F and 1G). When transected at region 8 (Figure 1F), the switch occurred between regions 5 and 6 on day 0. As regeneration proceeded, the switch site moved posteriorly toward the tail end, and by day 8, the switch was at region 8, near the original site of transection.

When transected at region 6 (Figure 1G), just posterior to the pharynx, the elongation/contraction switch was nearly the same as for the transection at region 8: the switch started at region 5/6 on day 0 and moved to region 7/8 by day 8. For all the transections, the location of the switch site on each day was statistically different from the site on all other days (*p* < 0.001, STAR Methods). Even though more of the worm had been removed in the region 6 transection, the switch site still moved the same amount back and changed its elongation/contraction switch site at a rate similar to the planarians cut at region 8 (Figure 1F). These results show that the same midbody region can act either as a head (Figures 1C–1E) or as a tail (Figures 1F and 1G) even though the various transections removed as much as 30% of the body length.

## Discussion

We were surprised that the sites of behavioral switching on either side of the transections change *immediately*, then slowly (in the course of a week or so) moves toward the site of transection as regeneration takes place. This suggests that there is an immediate change in the behavioral circuitry underlying interactions among the three behaviors, turning, elongation, and contraction. As regeneration progresses, with the addition of a significant amount of tissue, the physiological interactions apparently change in step with the anatomical changes to maintain coordination among the behaviors. For example, near-UV light stimulation produces turning in response to stimuli localized to roughly the anterior one-third of an intact worm. If that anterior one-third is removed, the same stimulus produces turning in the anterior one-third of the remaining piece of the worm. As the anterior end regenerates, the site of behavioral switching moves forward so that, at all times, the amount of the anterior end that produces turning remains roughly the anterior one-third of the regenerating worm.

A previous model^3^ suggested that there are front-to-back inhibitory connections of the nervous system that allow the anterior end of any piece of a planarian to act as the head. The current work adds two features to this model. First, the inhibitory effects appear to be local, within about 30% of the body length. Second, the inhibitory effects are plastic, with regeneration affecting the extent of the inhibition. In this way, the regional specificity of behaviors is maintained throughout regeneration. Because more tissue remodeling is necessary for smaller cut pieces and head regeneration is more complex than tail regeneration, as it must develop a new brain, worms cut at region 6 are not able to fully restore intact worm behavior by day 8, and much remodeling remains before the worm’s proportions are normal.

The completion of the local behavioral recovery that we have seen in this study is similar to the recovery of phototaxis^4^^,^^5^ after the head had been transected and aligns with the timeline of functional recovery of the neuronal network.^6^ A similar time course has been observed for the recovery of thermotaxis after head ablation.^7^ Our results show that regeneration works hand-in-hand with behavioral plasticity to keep the planarian functional as a major part of its body is being replaced.

### Limitations of the study

Our study is purely behavioral. It leads to hypotheses about possible physiological and molecular mechanisms but does not address them. Molecular techniques such as RNA interference^7^^,^^8^ have been worked out for planarians to study development, regeneration, and responses to drugs and toxic chemicals. In fact, planarians are the champions of regeneration: if cut into small pieces, they can regenerate a whole animal, including their cephalic ganglion.^9^ This extraordinary regenerative ability has been a major focus of researchers looking for mechanisms that might be used in more complex animals—such as humans—to enable them to regenerate lost body parts. In addition, the physiological model proposed could, in principle, be tested by electrically recording from the nervous system. Because of their small size and softness of their tissue, planarian electrophysiology is challenging.^10^ An alternative approach to recording neuronal activity is to incorporate voltage-sensing or Ca-sensing dye into neurons using molecular genetics. Such methods are not yet sufficiently developed in planarians to study the production and plasticity of behaviors. They would require a huge effort to address the issues raised by our studies.

A further limitation is that, because *D. japonica* breeds asexually, they cannot be used to study the influence of sex, gender, or both on behavior.

## Resource availability

### Lead contact

Requests for further information and resources should be directed to and will be fulfilled by the lead contact, William B. Kristan, Jr. (wkristan@ucsd.edu).

### Materials availability

This study did not generate new unique reagents.

### Data and code availability

• All data reported in this paper will be shared by the lead contact upon request.
• No new code was generated in this study.
• Any additional information required to reanalyze the data reported in this paper is available from the lead contact upon request.

## Acknowledgments

We thank Joyce Murphy for her able assistance in the UCSD Kristan laboratory, Dr. Kathleen French (UCSD) for many insightful discussions, William B. Kristan III (California State University, San Marcos) for crucial advice on the statistical analysis of the data, Gustav Allotey and Stephanie Liu (Swarthmore) for imaging regenerating planarians, and Dr. Danielle Ireland (Swarthmore) for feedback on the manuscript.

This research was supported in part by the 10.13039/100000001National Science Foundation under Grant NSF PHY-1748958 (W.B.K. Jr) and NSF CAREER Grant 1555109 (E.-M.S.C.)

## Author contributions

Conceptualization and methodology, W.B.K., Jr. and N.T.; investigation and formal analysis, N.T.; writing – original draft, W.B.K., Jr. and N.T.; writing – review and editing, N.T. and E.-M.S.C.; funding acquisition, W.B.K., Jr. and E.-M.S.C.; resources, W.B.K., Jr.; project administration, W.B.K., Jr.; supervision, W.B.K., Jr. and E.-M.S.C.

## Declaration of interests

The authors declare no competing interests.

## STAR★Methods

### Key resources table

**Table:** 

REAGENT or RESOURCE	SOURCE	IDENTIFIER
**Experimental models: Organisms/strains**		

*Dugesia japonica*	E-M Collins laboratory	N/A
*Dugesia japonica*	WB Kristan laboratory	N/A

**Statistics algorithms**		

R	R Studio IDE version 1.4.1106	https://rstudio.com
R	R version 4.1.0	https://cran.r-project.org

**Other**		

Near-UV laser pointer	Amazon	Anddicek™ near-UV (406 ± 10 nm)
Light power meter	Newport	Model 818 Photodiode Sensor

### Experimental model and subject details

For all of our experiments, we used the freshwater planarian flatworm *Dugesia japonica.* Their ancestors were originally obtained from Shanghai University, China and cultivated in the Collins laboratory for more than 10 years. They reproduce asexually, by pulling themselves into two pieces, with each half regenerating the missing half, forming two new worms. Given this means of reproduction, the age of the animals used is not a meaningful measure. For each experiment, we used the largest individuals available from our colony (1.0–1.5 cm while gliding), which were fully regenerated adults. The time to reach this size depends upon how much they eat at each feeding and their overall activity state, factors which we did not control. For each regeneration experiment in Figure 1, we used 16 animals, and we stimulated each one twice, for a total of 32 stimuli for each condition. No institutional permission and oversight for these animals was required because they are invertebrates.

We maintained the *D. japonica* in 3.8-L plastic Durable Food Storage containers (Kroger Food Store) filled with 1.5–2 L of planarian water maintained at room temperature (20-22°C). Initially, we made planarian water by adding 0.05g of Instant Ocean salts (Spectrum Brands, Blacksburg, VA, USA) per liter of DI water. Later, we used Crystal Geyser Natural Alpine Spring Water (from several different local grocery stores). We saw no difference in viability or behavior of the worms in these two conditions. We fed the planarians organic chicken liver (Whole Foods Market) which we froze and cut into 3–4 mm cubes once a week. Prior to their use in experiments, we did not feed them for 1–2 weeks. All experiments were conducted in planarian water, and we used each worm for only one set of regeneration experiments.

### Method details

We first used a Peltier cooling block (7 × 11 cm, Cambion, Cambridge MA) to anesthetize the planarians for surgical transection. We controlled the cooling block using a custom-built high-current 15V D.C. power supply. We ran tap water over the warm surface of the Peltier block and placed a planarian in a 35mm Petri dish containing planarian water on top of the cooled surface of the block. We squirted 95% ethanol between the bottom of the dish and the cooling block for good thermal contact. We used an electronic thermometer (Proster T-type) to measure the temperature of the planarian water. As the water cooled below 10°C, the planarians’ movements became slow; holding the temperature between 4°C and 6°C effectively anesthetized them. We used Dumont #5 forceps (Fine Science Tools, Foster City, CA) and a fine scalpel (15^o^ angle, 5.0 mm stab knife blade, Sharpoint Surgical Specialties, Reading, PA) to transect the planarians at different body locations. For all studies, we selected the largest planarians in our colony, which was 1.0 = 1.5cm.

Over the 8 days of the regeneration studies, we kept each experimental planarian in separate compartments of a 24-well Petri dish filled with planarian water. We transferred individual worms to a larger (35mm diameter x 10mm deep) Petri dish to perform each day’s experiment. We observed the planarian at 5X under a dissecting microscope. We tested each planarian within 5 min after the transfer. Each data point represents the average value from 16 individuals, with each planarian stimulated twice, waiting a minimum of 45 s between the two stimuli. We waited until the worm was gliding in a straight line before delivering the stimuli. We used a near-UV slit light stimulus to stimulate the worm in the different body regions using an Anddicek near-UV (406 ± 10 nm) laser pointer (Amazon). To produce the near-UV slits, we used the back end of a 10 mL plastic syringe cut across at about mid-length and placed over the end of the laser pointer. The unobstructed beam was 5 mm in diameter and produced a total power of 1.8mW (19 × 10^13^ photons/sec/mm^2^). To further localize the light stimulus, we put an opaque shield over the near-UV light source to admit a 0.5 mm by 5 mm slit of light (0.3mW total power; 25 × 10^13^ photons/sec/mm^2^). We used 16 animals selected rrandomly from among the largest (1.0–1.5 cm while gliding) in our colony, each stimulated twice each day, for the individual experiments. We repeated this procedure for each experimental worm over 8 days. The water in both the holding dishes and the experimental dishes were kept at 20-22°C.

#### Rationale for naming body regions

To measure how the sites of turn/elongate and elongate/contract switches change during regeneration, we needed to localize the stimuli. We first tried using “pre-pharyngeal”, “pharyngeal”, and “post-pharyngeal”, which are distinctions used in previous studies, but these regions turned out to be too crude to capture the changes we observed during regeneration. The reason for choosing to go with 11 body regions is partly convenience and partly functional. We tried dividing each of the 3 regions into 3 parts (pre-pharyngeal-anterior, pre-pharyngeal-middle, pre-pharyngeal-posterior, pharyngeal-anterior, etc.). But naming regions by their anatomical locations (e.g., PreA, PreM, PreP, PA, PM, PP, PostA, PostM, PostP) became cumbersome, confusing, and were of different lengths in different parts of the body. We found that numbering them sequentially in equal lengths starting at the front (Figure 1A) was more intuitive: Region 2, for instance was 20% of the way back from the anterior end of the intact animal and Region 6 was 60% of the way back. This system also turned out to be nicely correlated with behavioral switching: the turn/elongate switch was found to be always in Region 3 (Figures 1C–1E) and the elongate/contraction switch was always in Region 8 (Figures 1F and 1G).

The laser pointers used to deliver the near-UV slit stimuli were hand-held. Although this could potentially introduce errors, with practice, keeping the slit at 90° to the animal was not a problem (Figure 1A) and the regions are large enough that small hand movements did not affect the results. Localizing the stimulus to a single region was also relatively easy, because of the location and size of the pharynx. The pharynx occupies Regions 4 and 5, so those two regions are easy to differentiate as anterior (R4) and posterior (R5) regions over the pharynx. Regions 1–3 are also easily differentiated as the anterior (R1), middle (R2), and posterior (R3) regions between the auricles and the anterior end of the pharynx. Regions 6–10 are the most difficult to distinguish, but the most anterior region just posterior to the pharynx is R6, the most posterior region next to the tail is R10, so these regions had distinctive markers. That left R7-R9, which were readily identified as anterior (R7), middle (R8), and posterior (R9) regions between R7 and R10. To control for experimenter bias, a second person (WBK) performed some experiments on the same animals as the primary experimenter (NT); the localization of switching sites by the two experimenters was always identical.

### Quantification and statistical analysis

We used 16 animals for each of the experimental procedures (i.e., site of transection, direction of regeneration) and stimulated each animal piece twice each day for the 8 days of regeneration. Thus, number of animals for each data point is 16 and the number of stimuli is 32. These values are also given in the figure legend.

To evaluate the effects of cut location and days after transection on the site of behavioral switching, we tested whether the shift in behavioral response differed significantly between days for each of the cut locations. Because our data are not normally distributed and repeated observations of individuals are not independent, standard precision measures (e.g., mean, median, SD, SEM, confidence intervals) are not appropriate. Instead, we used mixed-effects Ordinal Logistic Regression to account for the ordered values of the locations that behavioral switches occurred along the planarian, while accounting for random effects of individual planarian variability. For conducting Ordinal Logistic Regression, we used R version 4.4.2 with the “clmm” function from the “ordinal” package to model the ordered nature of the behavior response. We used post-hoc Tukey tests to compare differences between days within each cut location condition, using the “emmeans” package. Statistical significance was set at *p* < 0.05 and a 95% confidence interval was used. We used location along the planarian as the ordinal response variable and day of regeneration as a numeric predictor. Cut location was a categorical predictor. The method looked at the interaction between day and cut location to evaluate whether the effect of day post-amputation varied across cut location conditions.

There is a significant interaction between day number and cut location, so we then did post-hoc comparisons between days within each cut location condition. Since day was used as a numeric variable, we specified the numeric values at which the post-hoc comparisons should be made, using day set to a value of 1, 2, 3, 4, 5, 6, 7, 8, and 9. Tukey comparisons of all different days against each other for each cut location condition were all significant with values of *p* < 0.0001. Hence, the switch location measured for each day after a given transection was strongly significantly different from the values for all other days. These data are also given in lines 195–211.
